# Factors Associated With Condom Breakage During Anal Intercourse: A Cross-Sectional Study of Men Who Have Sex With Men Recruited in an Online Survey

**DOI:** 10.2196/publichealth.5298

**Published:** 2016-02-22

**Authors:** Min Kim, Jennie McKenney, Christine M Khosropour, Adam B Prater, Eli S Rosenberg, Aaron J Siegler, Patrick S Sullivan

**Affiliations:** ^1^ Rollins School of Public Health Department of Epidemiology Emory University Atlanta, GA United States; ^2^ University of Washington Department of Medicine University of Washinton Seattle, WA United States; ^3^ Emory University Department of Radiology and Imaging Sciences University of Emory Atlanta, GA United States

**Keywords:** MSM, anal intercourse, condoms, condom failure

## Abstract

**Background:**

Within the United States, HIV affects men who have sex with men (MSM) disproportionally compared to the general population. In 2011, MSM represented nearly two-thirds of all new HIV infections while representing less than 2% of the US male population. Condoms continue to be the foundation of many HIV prevention programs; however, the failure rate of condoms during anal intercourse among MSM is estimated to be 0.5% to 8%, and condom breakages leave those affected at high risk for HIV transmission.

**Objective:**

Estimate the frequency of condom breakage and associated demographic and behavioral factors during last act of anal intercourse using data from a national online HIV prevention survey of MSM.

**Methods:**

From March 19 to April 16, 2009, data were collected on 9005 MSM through an online survey of US MSM recruited through a social networking site. Using multivariable logistic regression and controlling for several demographic and sexual risk behaviors, we estimated odds ratios of the association between condom breakage and several risk factors.

**Results:**

In the study, 8063 participants reported having at least one male sexual partner in the last 12 months. The median age of participants was 21 years (range 18-65). More than two-thirds (68.2%, 5498/8063) reported anal intercourse during last sex and 16.90% (927/5498) reported using a condom during last anal intercourse act. Condom breakage was reported by 4.4% (28/635) participants who engaged in receptive anal intercourse and 3.5% (16/459) participants who engaged in insertive anal intercourse, with an overall failure rate of 4.0% (95% CI 3.2%-6.0%). Age (adjusted odds ratio [aOR] per 5 years: 0.53 (95% CI 0.30-0.94), number of male sex partners (aOR per 5 sex partners: 1.03 (95% CI 1.00-1.08), and being high or buzzed during sex with a casual sex partner (aOR: 3.14, 95% CI 1.02-9.60) were associated with condom breakage.

**Conclusions:**

Our results indicate condom breakage is an important problem for MSM that may be more common for younger men, for men with more partners, and during sex with casual partners after alcohol consumption or drug use. A better understanding of why condom breakage occurs more often in these groups is needed to improve educational efforts. Further, during this time of expanded interest in new condom designs, consideration should be given to how condom design might minimize breakage during anal sex.

## Introduction

Men who have sex with men (MSM) continue to be disproportionately affected by HIV/AIDS in the United States. MSM represent less than 2% of the male population in the Unites States, but male-to-male sexual contact remains the predominant mode of HIV transmission, accounting for an estimated 65% of all new HIV diagnoses in 2011 [1,2]. Further, since 2008, the proportion of HIV diagnoses attributable to male-to-male sexual contact and the rate of HIV transmission among MSM continued to increase while trends for other transmission categories and groups have remained the same or declined [1-3]. The high prevalence of condomless anal intercourse among MSM coupled with the fact that anal intercourse is associated with greater HIV transmission probabilities compared to vaginal intercourse provides some explanation of the large burden of disease experienced among MSM [4-9].

Despite suboptimal utilization, male condoms have been and remain a constant in HIV prevention programs due to their effectiveness in reducing transmission of HIV/STIs when used correctly and consistently [10-17]. To date, however, the US Food and Drug Administration has only cleared condoms for use during vaginal intercourse and has warned against the use of condoms during anal intercourse [18]. Because of the physiological differences between anal intercourse and vaginal intercourse, such as friction and compression, it is possible that condoms break differentially by application [19-20].

Condom failure, defined as breakage or slippage of a condom during intercourse, can obviate the prevention benefit of condom usage, but data on condom failure rates and condom failure during specific sex acts have produced a wide range of failure rates [10,21-28]. In two separate studies, D’Anna et al reported higher rates of condom breakage or slippage during vaginal intercourse among heterosexual couples (6%) compared to anal intercourse among MSM couples (3%) [22,23]. Other studies have shown similarly low rates of condom failure (per condom use) among those engaging in anal intercourse (2%-3%), compared to vaginal intercourse [10,24-26].

Data on predictors of condom failure among MSM is limited. Penile length and circumference, absence of lubricants, race, and lower socioeconomic status have all been associated with higher rates of condom failure [29,30]. Further identification of behaviors associated with condom failure is key in order to identify high-risk groups and behaviors that would benefit from targeted condom education. Using data from a national online HIV prevention survey of MSM, we aimed to document how often condom breakage was reported to occur during anal intercourse between MSM and to identify demographic and behavioral characteristics associated with condom breakage during an act of anal intercourse.

## Methods

### Recruitment and Ethics

We utilized data from the Barriers to Online Prevention Research survey of US MSM collected between March 19, 2009 and April 16, 2009. The methods have been previously reported [31]. Briefly, participants were recruited from MySpace, a large social networking site, using banner advertisements. Advertisements were directed at male users of MySpace18 years or older who resided within the United States. Once the banner advertisement was clicked, individuals were screened for eligibility and provided informed consent. Eligible participants included men 18 years or older who had at least one male sex partner in the past year and were residents of the United States. Eligible men were then asked to complete a Health Insurance Portability and Accountability Act-compliant online survey on SurveyGizmo (Boulder, CO). The survey took approximately 30 to 45 minutes to complete, and no compensation was provided to participants. The study protocol was approved by the Emory University Institutional Review Board.

### Data Collection

Participants were asked a series of questions regarding demographics, sexual history, most recent sex act, and most recent sexual partner. Specifically, participants were asked to report the number of male sexual partners in the last 12 months and if they were high or buzzed during last act sex act. Participants were also asked questions pertaining to their most recent sex partner, such as if he was a main or casual partner. A main partner was defined as someone that the participant felt committed to above all others, and a casual partner was defined as one whom the participant did not feel committed to above all others. With regard to their last sexual partner, participants were asked whether they engaged in anal and/or oral sex with him, if a condom was used, and if the condom broke during last anal intercourse. A copy of the survey items relevant to the present study can be seen in Multimedia Appendix 1.

Our outcome measure, condom breakage, was assessed via a categorical response to two separate questions designed to capture both receptive and anal insertive sex acts. Men were first asked if they had receptive anal intercourse, insertive anal intercourse, or both during their last sex episode. Based on their responses, men were asked separately about condom use when they were the receptive and/or insertive partner. Questions were: “Did [your last sexual partner] use a condom the last time you had *receptive* anal sex?” and “Did you use a condom the last time you had *insertive* anal sex?” Responses for both questions included “He (I) did not use a condom,” “He (I) used a condom part of the time,” “He (I) used a condom the whole time,” “He (I) used a condom but it broke,” “Don’t know,” or “Prefer not to answer.” Condom breakage was defined as answering “He (I) used a condom but it broke” for either insertive or receptive anal sex acts with the last male sex partner of the participant.

### Data Analysis

Data were analyzed using SAS version 9.4 (SAS Institute). Participants included in the final analysis reported engaging in receptive or insertive anal intercourse with their last male partner in the past 12 months and using a condom during last anal intercourse. Bivariate analyses were conducted to examine unadjusted correlates of condom breakage. For continuous variables, a Wilcoxon rank-sum *z* test (two-sided) was used due to the nonnormally distributed nature of the variables. Statistically significant covariates (*P* value ≤.05) were included in the final model, as were variables found to be associated with condom breakage in previous studies [20-22,25,32,33].

The dependent variable, condom breakage, was calculated as a proportion and modeled the event level for both receptive and insertive anal intercourse at last sex. Thus, one participant could contribute two observations (one for receptive and one for insertive sex) from his last sexual episode. Multivariable analysis was conducted using multivariable logistic regression controlling for repeated observations. Odds ratios and 95% confidence intervals were calculated for categorical variables. For continuous variables, odds ratios and confidence intervals per unit of 5 were calculated.

## Results

**Figure 1:**
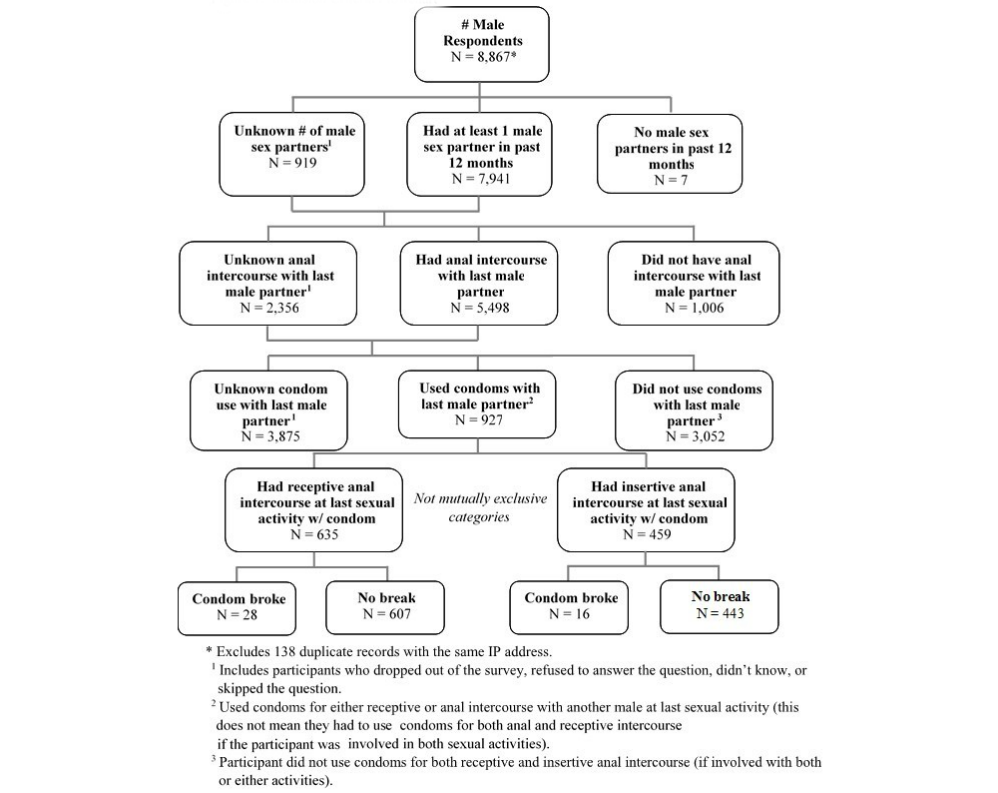
Inclusion criteria flowchart.

A total of 9005 participants completed the initial screening questions and gave consent; 133 surveys were linked to duplicate IP addresses and were therefore excluded. Of the 8872 unique surveys completed, 62.0% (5498/8872) were completed by men who reported anal intercourse at last sex; 44.0% (3875/8872) did not know if they used a condom at last anal intercourse. A total of 10.4% (927/8872 of the surveys were completed by participants who reported condom use at last sex and thus were eligible for multivariable analysis. Figure 1 describes how participants were classified and identified for inclusion in the analysis.

Demographic information on the 927 participants who reported using a condom at last anal intercourse and the 3052 participants who reported not using a condom at last anal intercourse is provided in Table 1.

**Table 1:** Demographic and behavioral characteristics of men at last act of anal intercourse with a male sexual partner, stratified by condom use, among participants of an online HIV prevention survey, United States, March-April 2009.

Characteristics		Used condoms^a^ (N=927) n (%)	Did not use condoms^a^ (N=3052) n (%)	Total(N=3979) n (%)
**Race/ethnicity** ^b^				
	Black/African-American	150 (16.3)	354 (11.7)	504 (12.8)
	Latino/Hispanic	365 (39.8)	962 (31.8)	1327 (33.7)
	White/Caucasian	281 (30.6	1375 (45.5)	1656 (42.0)
	Other^c^	122 (13.3)	331 (11.0)	453 (11.5)
**Education**				
	≤ High school diploma/GED	394 (43.4)	1269 (42.0)	1663 (42.3)
	> High school diploma/GED	516 (56.7)	1752 (58.0)	2268 (57.7)
Age (years), median (range)^b^		21.0 (18-65)	22.0 (18-65)	21.0 (18-65)
**Sexual identity** ^b^				
	Heterosexual/straight	669 (72.6)	2366 (78.2)	3035 (76.9)
	Homosexual/gay	236 (25.6)	628 (20.8)	864 (21.9)
	Other^d^	17 (1.8)	32 (1.1)	49 (1.2)
Number of male sex partners in last 12 months, median (range)^b^		5.0 (1-365)	3.0 (1-365)	4 (1-365)
**Type of MRMSP** ^b^				
	Main	448 (52.1)	2039 (71.1)	2487 (66.7)
	Casual	412 (47.9)	829 (28.9)	1241 (33.3)
**Race/ethnicity of MRMSP** ^b^				
	Black/African-American	157 (17.2)	360 (12.0)	517 (13.2)
	Latino/Hispanic	298 (32.7)	793 (26.4)	1091 (27.9)
	White/Caucasian	366 (40.2)	1592 (53.1)	1958 (50.1)
	Other^c^	90 (9.9)	256 (8.5)	346 (8.8)
**Racial concordance with MRMSP** ^e^				
	Yes	500 (55.4)	1695 (56.9)	2195 (56.5)
	No	403 (44.6)	1286 (43.1)	1689 (43.5)
Age of MRMSP, median (range)^b^		23 (18-70)	24 (18-70)	23 (18-70)
**Age discrepancy with MRMSP**				
	Participant is at least 5 years younger	232 (25.0)	740 (24.3)	972 (24.4)
	Participant is within 5 years in age	612 (66.0)	2045 (67.0)	2657 (66.8)
	Participant is at least 5 years older	83 (9.0)	267 (8.8)	350 (8.8)
**MRMSP is an exchange partner** ^f^				
	Yes	28 (3.1)	64 (2.1)	92 (2.3)
	No	888 (96.9)	2977 (97.9)	3865 (97.7)
**High or buzzed during sex** ^g^				
	Yes	225 (25.3)	681 (23.2)	906 (23.7)
	No	665 (74.7)	2259 (76.8)	2924 (76.3)
**HIV status of MRMSP**				
	HIV positive	32 (5.3)	116 (5.0)	148 (5.1)
	HIV negative	577 (94.8)	2186 (95.0)	2763 (94.9)

^a^Column percentages may not add up to 100% due to rounding; missing values were not included.

^b^ *P* value ≤.05.

^c^Other races include Asian/Pacific Islander, Native American/Alaskan Native, multiple, and other races.

^d^Other sexual identities include bisexual and other.

^e^A participant is racially concordant with MRMSP if he/she reports the same race/ethnicity as the MRMSP.

^f^Exchange partner is defined as a partner with whom the participant had sex in exchange for things they needed (eg, money, drugs, food, shelter, or transportation).

^g^Includes being high or buzzed with alcohol, drugs not prescribed by a doctor, or both during sex.

Most participants were members of a racial or ethnic minority: 33.7% (1327/3979) were Hispanic, 12.8% (504/3979) were black non-Hispanic, and 11.5% (453/3979) were multiracial. Most reported having attended some college, and three-quarters of all participants were aged 18 to 26 years. The median number of male sex partners in the past 12 months was 3, and over half of participants’ last male sex partners were casual partners. Nearly 20% (906/3979) of participants had used drugs or alcohol prior to engaging in sex at last sexual episode, and almost 2% (92/3979) of participants reported exchange sex with their most recent male sex partner.

Of the 927 participants included in the final analysis, 69.0% (635/927) reported using a condom during receptive anal intercourse and 50.0% (459/927) reported using a condom during insertive anal intercourse. Overall, condom breakage was reported in 4.0% (44/1094 , 95% CI 3.2%-6.0%) of the total distinct episodes of anal intercourse. Condom breakage was reported by 4.4% (28/635) participants who engaged in receptive anal intercourse and 3.5% (16/459) participants who engaged in insertive anal intercourse.

Results from the multivariable analysis are presented in Table 2. Younger age and being buzzed or high before or during sex with a casual partner were associated with condom breakage during last anal intercourse, while number of male sexual partners in the past year was marginally significant. Participants’ odds of condom breakage increased 3% for every 5 male sex partners reported in the past year (adjusted odds ratio 1.03, 95% CI 1.00-1.08). Participants who reported being high or buzzed at last sex with a casual sex partner had 3 times the odds of condom breakage compared to participants who reported being high or buzzed at last sex with a main partner (95% CI 1.02-9.6). The odds of condom breakage were 0.53 for every 5-year increase in age of a participant (95% CI 0.30-0.94).

**Table 2:** Associations between demographic and behavioral factors and condom breakage among men who had anal intercourse with their last male sex partner, in an online HIV prevention survey, United States, March-April 2009.

Characteristics		Broken condom^ab^ (N=41) n (%)	No broken^ab^condom (N=881) n (%)	Crude odds ratio (95% CI)	Adjusted odds ratio (95% CI)
**Race/ethnicity**					
	Black/African-American	10 (7)	139 (93)	1.95 (0.79-4.80)	2.13 (0.79-5.77)
	Latino/Hispanic	13 (4)	349 (96)	1.01 (0.4-2.34)	0.78 (0.29-2.08)
	Other^c^	8 (7)	113 (93)	1.92 (0.74-4.99)	1.98 (0.6-5.76)
	White/Caucasian	10 (4)	271 (96)	Referent	Referent
**Education**					
	≤ High school diploma/GED	19 (5)	374 (95)	1.32 (0.69–2.53)	0.84 (0.39-1.81)
	> High school diploma/GED	19 (4)	493 (96)	Referent	Referent
Age (year), median (range)^d^		20 (18-47)	21 (18-65)	0.69 (0.48-1.01)	0.53 (0.30-0.94)
**Number of male sex partners in last 12 months** ^d^		6 (1-364)	4 (1-87)	1.05 (1.02-1.07)	1.03 (1.00-1.08)
**MRMSP is an exchange partner** ^e^					
	Yes	4 (14)	24 (86)	3.93 (1.29-11.91)	0.63 (0.06-6.31)
	No	36 (4)	848 (96)	Referent	Referent
**Type of MRMSP**					
	Main	23 (5)	421 (92)	1.45 (0.74-2.81)	See interaction
	Casual	15 (4)	397 (96)	Referent	See interaction
**High or buzzed during sex** ^f^					
	Yes	16 (7)	209 (93)	2.33 (1.20-4.56)	See interaction
	No	21 (3)	640 (96)	Referent	See interaction
**Interaction, high or buzzed during sex**					
	Casual partner	10 (9)	107 (91)	―	3.14 (1.02-9.60)
	Main partner	4 (5)	82 (95)	―	Referent
**Interaction, not high or buzzed during sex**					
	Casual partner	4 (1)	274 (99)	―	0.31 (0.08-1.27)
	Main partner	17 (5)	324 (95)	―	Referent

^a^Column percentages may not add up to 100% due to rounding; missing values were not included.

^b^Condom break is defined as a break in the condom during either insertive or receptive anal intercourse (or both) during last sexual activity with a male partner. No condom breakage is defined as no break in the condom at last sexual activity (with both insertive and receptive anal intercourse) with a male partner.

^c^Other races include Asian/Pacific Islander, Native American/Alaskan Native, multiple, and other races.

^d^Per 5-unit increase.

^e^Exchange partner is defined as a partner with whom the participant had sex in exchange for things they needed (eg, money, drugs, food, shelter, or transportation).

^f^Includes being high or buzzed with alcohol, drugs not prescribed by a doctor, or both during sex.

## Discussion

### Principal Findings

Results from our multivariable analysis of condom breakage among US MSM revealed an overall condom breakage rate of 4%. Condom breakage rates did not differ between participants who reported receptive and insertive anal intercourse. Younger age, a greater number of sexual partners reported in the last 12 months, and being buzzed or high at last sex with a casual partner were associated with condom breakage.

The overall condom breakage rate of 4% is in line with previous studies [10,14-18], but there is considerable variability among breakage rates from prior studies [33-40]. Golombok et al found a condom failure rate of 2% in a group of 283 homosexual couples in the United Kingdom, but the study focused on sexual activity among long-term couples, which does not represent the MSM population [19]. Our sample was younger, and more than half of sex partners were reported to be casual partners. A 6-month condom breakage risk of 31% was found in a cohort study of MSM in Atlanta, GA [35]. The high frequency of condom breakage found in the aforementioned Atlanta cohort study compared to other studies is likely due to the longer recall period (6 months), allowing for a higher number of sex acts to occur. Further, the study revealed 40% of black MSM reported breakage or incomplete use; the population was twice as likely to report condom breakage as white MSM. Similar point estimates were seen from our analysis; however, our sample size was not sufficient to assess the relationship. The wide variability of condom failure rates among these studies is most likely a reflection of the diverse population of MSM and sample sizes under study [36-40].

For every 5 male sexual partners, we found that the odds of condom breakage increased by 3%. There are likely user characteristics of participants with large numbers of sexual partners not captured by our survey that explain the statistical association with condom breakage. Participants with a larger number of male sexual partners might have engaged in more aggressive coital behaviors than those with fewer sexual partners, leading to greater stress on the condom [41,42]. Further, participants with a large number of male sexual partners may have a predisposition to inappropriately use lubricant, resulting in condom failure [43]. MSM who report high numbers of sexual partners represent a risk group commonly targeted for behavioral interventions, as having multiple sexual partners is an established risk factor for HIV acquisition [38]. Results from our study suggest that behavioral prevention interventions targeted to this high-risk group should also include more thorough condom education.

Several studies demonstrated that drug and alcohol use is associated with an increased risk of HIV acquisition among heterosexual men engaging in vaginal intercourse and MSM engaging in anal intercourse [44,45]. Alcohol use during sex is higher among casual partners compared to main partners; however, few studies have assessed the association between condom failure and partner type, modified by alcohol and drug use before or during sex [46,47]. Results from our study suggest that being high or buzzed during sex with a casual partner was associated with increased odds of condom breakage compared to being high or buzzed with a main partner. Alcohol and drug use may prolong ejaculation and thereby prolong sex, increasing the risk of condom failure [27]. Moreover, alcohol and drug use may impede the proper application and usage of condoms, increasing condom failure rates [27]. These results suggest that public health prevention interventions need to increase awareness of the effects of drug and alcohol use during sex, specifically targeting MSM who engage in sex with casual partners.

### Limitations

There are several limitations to this study. Most notably, our respondents are not representative of all MSM in the United States. Due to the nature of the survey, participants had to be proficient with computers, making them more likely to have a higher education and income than the general population of MSM. Our study used a cross-sectional study design to evaluate condom breakage at last intercourse and therefore did not capture condom use or breakage over time. Safe sex behaviors fluctuate over time [31], making a prospective study more appropriate to capture the time-dependent nature of condom use and failure. We did not ascertain a history of lubrication use or lubrication use during last sex act. Therefore, our sample may represent men who were less likely to use lubrication, and thus, factors associated with condom breakage may actually be factors associated with lubrication use. The majority of studies that have evaluated condom failure define it as condom breakage or slippage during sex [36-39]. Our study only used condom breakage as a measurement of condom failure. Omission of condom slippage data resulted in our inability to report condom failure more generally. Our study uses older data; however, our finding of a condom breakage rate of 4% is in line with previous and more recent studies [10,14-18]. Finally, responses may have been affected by social desirability bias and recall bias, resulting in misclassification of outcomes [48].

### Conclusion

Results from our study highlight condom breakage rates among a diverse sample of MSM from the United States. While condoms remain a strong component of prevention policy, our findings indicate condoms may not suffice as a sole means of reducing HIV transmission risk within the context of a high per-act transmission risk of anal sex [6]. Combining condoms with other prevention modalities such as preexposure prophylaxis can help mitigate risk that occurs after condom breakage [49]. Even in the context of preexposure prophylaxis, more efforts should be made to improve condom use practices that might lead to condom failure [49]. MSM who report a high number of sexual partners as well as those who use drugs and/or alcohol before or during sex with a casual partner are at an increased risk for condom failure and would benefit from targeted condom education programs to help mitigate their risks. As innovation continues to occur in the design of condoms, it will be important to consider if there are new types or designs of condoms that might decrease condom breakage during anal intercourse [49]. To best understand when, why, and how condom failures occur, further studies are needed to capture the time-dependent nature of condom use; these should include high-risk sexual behaviors; precoital factors, such as condom storage; and coital factors, such as duration of sex act and use of lubricants. As HIV continues to disproportionately affect United States MSM and anal intercourse remains a significant mode of HIV transmission, condom failure among this population needs to be addressed through multiple approaches.

## Appendix

Multimedia Appendix 1 Barriers to Online Prevention Survey.

## Abbreviations

MSM: men who have sex with men
