# Supplementary material for: Factors Associated With Condom Breakage During Anal Intercourse: A Cross-Sectional Study of Men Who Have Sex With Men Recruited in an Online Survey
Source: JMIR Public Health Surveill. 2016 Feb 22;2(1):e7. doi: 10.2196/publichealth.5298 (PMC4869233; doi:10.2196/publichealth.5298)
Supplement: Multimedia Appendix 1 [file publichealth_v2i1e7_app1.pdf]

Appendix 1. Barriers to Online Prevention Survey

Health Survey

Demographics

Thank you for interest in our survey.

Helpful tips:

- **Please note that at any time during the survey, you can save your progress and return later to complete the survey -- just click the text** "Save and continue survey later" **on the top right of the browser window.**
- **Questions marked with a red asterisk (*) are required questions that you must answer to move forward.**
- **Use the back button at the bottom of each page, rather than the back button on your browser.**

First, we have a few questions to determine if you are eligible to participate in the survey.

1) What is your sex?*

( ) Male

( ) Female

2) What is your age?*

____

3) In the past 12 months, have you had sex with:*

( ) One or more men

( ) One or more women

( ) Both men and women

( ) I have not had sex in the past year

Race and sexual orientation

5) Do you consider yourself to be Hispanic or Latino?

( ) Yes

( ) No

( ) Don't Know

( ) Prefer not to answer

6) Which racial group do you consider yourself to be in?

( ) Asian/Pacific Islander

( ) Black/African-American

( ) White/Caucasian

( ) Native American/Alaska Native

( ) Multi-Racial

( ) Prefer not to answer

( ) Other: ____

You indicated that you are multiracial. Please check all of the racial groups your consider yourself to be in.

[ ] Asian/Pacific Islander

[ ] Black/African American

[ ] White/Caucasian

[ ] American Indian/Alaskan Native

[ ] Other: ____

7) What is your zip code?

____

Education and identity

8) What is the highest grade in school you completed?

( ) College, post graduate, or professional school

( ) Some college, Associate's degree, and/or Technical school

( ) High school or GED

( ) Some high school

( ) Less than high school

( ) Never attended school

( ) Don't Know

( ) Prefer not to Answer

9) Do you think of yourself as:

( ) Heterosexual or "Straight"

( ) Homosexual, Gay

( ) Bisexual

( ) Other: ____

( ) Prefer not to answer

Outness and venue attendance

10) In the last 12 months, how often have you gone to a bar or dance club frequented by gay men?

( ) Never attend

( ) Once a month or less

( ) About once a week

( ) Several times a week

( ) Once a day or more

( ) Don't know

( ) Prefer not to answer

11) In the last 12 months, how often have you gone to a bathhouse or sex club frequented by gay men?

( ) Never attend

( ) Once a month or less

( ) About once a week

( ) Several times a week

( ) Once a day or more

( ) Don't know

( ) Prefer not to answer

Online sex seeking

12) In the past 12 months, have you gone online to meet sex partners?

( ) Yes

( ) No

( ) Prefer not to answer

How did you try to meet sex partners online? Check all that apply.

[ ] Through online personal ads

[ ] Through online chatrooms

[ ] By using online communities or message boards

[ ] By visiting web sites that are free of charge

[ ] By visiting websites that require a paid subscription

[ ] Other: ____

Sexual activities

The following questions are about sexual activity. Remember that all of the information you share is confidential, and we don't want to know your name. It's important that your answers be as accurate as possible.

13) During the past 12 months, how many different *men* have you had anal or oral sex with? Estimates are OK if you don't remember the exact number.*

____

14) During the past 12 months, how many different *women* have you had vaginal or anal sex with? Estimates are OK if you don't remember the exact number.*

____

Male partner types

This question asks about types of sex partners. A main partner is someone that you feel committed to above all others -- this is someone you might call your boyfriend, significant other, life partner, or husband. A casual partner is someone that you do not feel committed to above all others.

15) Of the [%%45:%%] male partners you had sex with in the past 12 months, how many were:

________main partners?

________casual partners?

16) Was any of these [%%45:During the past %%] male sex partners an exchange partner -- that is a partner that you have sex with in exchange for money, drugs, food, or something else of value?

( ) Yes

( ) No

( ) Don't know

( ) Prefer not to answer

Male AI proportions

17) Of the [%%45: During the past %%] male partners you had anal or oral sex with in the past 12 months, how many did you meet on the Internet?

____

18) Of the [%%45:During the past %%] male partners you had anal or oral sex with in the past 12 months, how many did you have anal sex with?

____

Male proportion UAI COPY

19) Of your [%%49: Of the [%% %%] partners you had anal sex with in the past 12 months, how many did you have unprotected anal sex with? (This means that you or your partner did not use a condom at any time during sex).

____

20) In the past 12 months, did you have unprotected anal sex with your male anal sex partner? (This means that you or your partner did not use a condom at any time during sex).

( ) Yes

( ) No

( ) Don't know

( ) Prefer not to answer

Main Partner

The following questions are about your most recent male sex partner. This is the last man that you had sex with, and could be your main sex partner or a casual sex partner. By sex, we mean either oral or anal sex.

21) Please enter a nickname for this partner to help make the questions clearer. This should NOT be his real name – it could be his initials, or a nickname that you call him. This name will NOT be saved in your responses; it is just to help make the next few questions clearer.

____

SEXFREQ MAIN MALE

22) In what month and year did you *first* have sex with [%%54: Please enter a %%]?

23) In the past year, how many times have you had anal or oral sex with [%%54: Please enter a %%]?

( ) One time

( ) 2 - 5 times

( ) 6 - 10 times

( ) More than 10 times

( ) I don't know

( ) Prefer not to answer

About how often did you have anal or oral sex with [%%54: Please enter a %%] over the past year?

( ) About once a month

( ) 2 or 3 times a month

( ) About once a week

( ) 2 or 3 times a week

( ) More than 3 times a week

24) Have you had anal sex with [%%54: Please enter a %%] in the past 12 months?

( ) Yes

( ) No

( ) Don't know

( ) Prefer not to answer

Have you had unprotected anal intercourse with [%%54: Please enter a %%] in the past 12 months? This means that you or your partner did not use a condom at all during anal sex.

( ) Yes

( ) No

( ) Don't know

( ) Prefer not to answer

About main male partner

Now we have a few questions about [%%54:%%].

25) Where did you first meet [%%54: Please enter a %%]?

( ) Bar/Club

( ) Cruising area

( ) Adult bookstore

( ) Bath house, sex club or sex resort

( ) Private sex party

( ) Circuit party or Rave

( ) Internet

( ) On the street

( ) Through friends

( ) At church

( ) School or work

( ) Through a personal ad in a newspaper

( ) On a telephone chat line or dating line

( ) Other: ____

26) What is [%%54: Please enter a %%]'s current age?

As far as you know, which of the following statements about [%%54: Please enter a %%]'s age is most true?

( ) He is within a year of my age

( ) He is at least 2 years younger than I am

( ) He is 2-10 years older than I am

( ) He is more than 10 years older than I am

27) As far as you know, does [%%54: Please enter a %%] consider himself to be Hispanic or Latino?

( ) Yes

( ) No

( ) Don't know

( ) Prefer not to answer

28) As far as you know, what race does [%%54: Please enter a %%] consider himself to be?

( ) Asian/Pacific Islander

( ) Black/African-American

( ) White/Caucasian

( ) Native American/Alaska Native

( ) Multi-Racial

( ) Decline to Respond

( ) Other: ____

29) What kind of sex partner is [%%54:Please enter a %%]?

( ) A main sex partner (Someone you feel committed to above all others)

( ) A casual sex partner (Someone you do not feel committed to above all others)

30) Is [%%54: Please enter a %%] an exchange partner (someone who you have sex with in exchange for money, drugs, food, or something else of value)?

( ) Yes

( ) No

( ) Don't know

( ) Prefer not to answer

Last sex question

The next questions are about the last time you had sex with [%%54:%%]. Remember, your answers are confidential.

31) In what month and year did you *most recently* have sex with [%%54: Please enter a %%]?

32) The last time you had sex with [%%54: Please enter a %%], did you have receptive anal sex? (This means that you were the bottom)

( ) Yes

( ) No

( ) Don't know

( ) Prefer not to answer

Did [%%54: Please enter a %%] use a condom the last time you had receptive anal sex (bottomed)? Choose one.

( ) He did not use a condom

( ) He used a condom part of the time

( ) He used a condom the whole time

( ) He used a condom, but it broke

( ) Don't Know

( ) Prefer not to Answer

33) The last time you had sex with [%%54: Please enter a %%], did you have insertive anal sex? (This means that you were the top).

( ) Yes

( ) No

( ) Don't know

( ) Prefer not to answer

Did you use a condom the last time you had insertive anal sex with [%%54: Please enter a %%]? Choose one.

( ) I did not use a condom

( ) I used a condom part of the time

( ) I used a condom the whole time

( ) I used a condom, but it broke

( ) Don't Know

( ) Prefer not to Answer

Last Sex Situation

34) The last time you had anal or oral sex with [%%54: Please enter a %%], were you high or buzzed on any of the following?

( ) Alcohol

( ) Drugs not prescribed by a doctor

( ) Both alcohol and drugs not prescribed by a doctor

( ) Neither drugs nor alcohol

( ) Don't Know

( ) Prefer not to Answer

35) The last time you had anal or oral sex with [%%54 Please enter a %%], did you know his HIV status?

( ) Yes

( ) No

( ) Don't know

( ) Prefer not to answer

The last time you had sex with [%%54: Please enter a %%], what was his HIV status?

( ) HIV-negative

( ) HIV-positive

( ) Prefer not to Answer

Knowledge of HIV status before first sex male partner

36) Before you had sex with [%%54: Please enter a %%] *for the first time in [%%264:firstsexmo %%] [%%191:Year %%],* **did you discuss BOTH your HIV status AND his HIV status?**

( ) Yes

( ) No

( ) Don't know

( ) Prefer not to answer

/Inconclusive

( ) Didn't get the results of my last HIV test

( ) Prefer not to Answer
